# Child and maternal health outcomes following antenatal exposure to classic psychedelic substances: a systematic review

**DOI:** 10.21203/rs.3.rs-9732799/v1

**Published:** 2026-05-19

**Authors:** Sunjuri Sun, Claudia Hanson, Peter S. Hendricks, Simon B. Goldberg, Karilynn M. Rockhill, Adhithi Sreenivasan, Lena Elise Wessing, Mary Hewitt, Paul Liknaitzky, Walter Osika, Jayanth Narayanan, Otto Simonsson

**Affiliations:** Department of Global Public Health, Karolinska Institutet, Stockholm, Sweden; Department of Global Public Health, Karolinska Institutet, Stockholm, Sweden; London School of Hygiene and Tropical Medicine, London, United Kingdom; Department of Psychiatry and Behavioral Neurobiology, University of Alabama School of Medicine, Birmingham, USA; Department of Counseling Psychology, University of Wisconsin, Madison, USA; Center for Healthy Minds, University of Wisconsin–Madison, Madison, WI; Rocky Mountain Poison and Drug Safety, Denver Health and Hospital Authority, Denver, Colorado; Department of Global Public Health, Karolinska Institutet, Stockholm, Sweden; Department of Global Public Health, Karolinska Institutet, Stockholm, Sweden; Department of Global Public Health, Karolinska Institutet, Stockholm, Sweden; Department of Psychiatry, Monash University, Australia; School of Psychological Sciences, Monash University, Australia; Department of Neurobiology, Care Sciences and Society, Karolinska Institutet, Stockholm, Sweden; Department of Management & Organizational Development, Northeastern University, Boston, MA, USA; Department of Neurobiology, Care Sciences and Society, Karolinska Institutet, Stockholm, Sweden

**Keywords:** Obstetrics, Population Health, Psychedelics, Pregnancy, Maternal and Child Outcomes, Systematic Review, Perinatal Outcomes, Prenatal Exposure

## Abstract

**Importance:**

As the cultural, scientific, and legal landscape surrounding psychedelics continues to shift toward greater access and acceptability, it is likely that increasing numbers of women of reproductive age may be exposed to these substances. Despite this, the evidence on antenatal exposure has not been systematically reviewed.

**Objective:**

To synthesize the human literature on child and maternal outcomes following antenatal exposure to classic psychedelics.

**Evidence review:**

A systematic literature search was conducted in Medline, Embase, Web of Science, PsycInfo and CENTRAL for original peer-reviewed studies published from inception to 15 July, 2025, that reported on antenatal exposure to psychedelics. Two independent reviewers conducted title and abstract screening, full text screening, data extraction and risk of bias assessment. The Joanna Briggs Institute (JBI) critical appraisal tools were used for risk of bias assessment. As significant heterogeneity in outcome reporting and study design precluded meta-analysis, the findings were qualitatively synthesized. The protocol was pre-registered on PROSPERO (CRD420251082910).

**Findings:**

A total of 42 studies met the inclusion criteria, encompassing 251 women who were exposed to a psychedelic substance during pregnancy or prior to pregnancy from across 9 countries. Almost all studies were conducted in high-income countries (k = 38, 90.5%), of which most were conducted in the US (k = 29, 69.0%). LSD was the most commonly reported psychedelic substance (k = 38, 90.5%), more than half of the articles were case reports (k = 23, 54.8%), and most studies were published prior to the year 2000 (k = 31, 73.8%). In terms of child and maternal outcomes, two articles (4.8%) reported on the outcome of spontaneous abortions, no articles reported on maternal deaths, one (2.4%) on stillbirth, 16 (38.1%) on neonatal mortality, 17 (40.5%) on preterm birth, 15 (35.7%) on birthweight and 26 (61.9%) on congenital malformations. The certainty of evidence for all outcomes was deemed to be very low using the GRADE approach.

**Conclusions and relevance:**

The current literature on child and maternal outcomes following antenatal exposure to psychedelics is notably sparse, mainly consisting of case reports or small sample sizes drawn almost entirely from high-income countries. These findings highlight that contemporary, methodologically robust research on psychedelics during pregnancy is urgently needed.

## Introduction

The use of classic psychedelics such as psilocybin and lysergic acid diethylamide (LSD) has increased in the US in recent years.^1,2^ For example, from 2019 until 2023, past-year use of psilocybin in the United States (US) increased 44% among young adults and 188% among older adults.^2^ This increase has coincided with renewed scientific interest in psychedelics as treatments for psychiatric disorders,^3^ changing perceptions of safety among the public,^4^ and state-level decriminalization and legalization initiatives,^5^ as well as US Food and Drug Administration (FDA) breakthrough therapy designations for psychedelics in the treatment of mood disorders such as postpartum depression.^6,7^ Together, these trends suggest that the likelihood that women of reproductive age will be exposed to these substances during pregnancy may be increasing.

Despite this trajectory, remarkably little is known about the effects of psychedelic exposure during pregnancy on child and maternal health outcomes.^8^ Furthermore, clinical trials exclude pregnant individuals due to ethical and safety concerns. Naturalistic use of psychedelics during pregnancy has also been documented amongst certain populations for cultural or religious reasons, such as ayahuasca consumption amongst the Santo Daime community in Brazil and peyote use in the Native American Church (NAC).^9–11^ Given the need to provide accurate counselling to women of reproductive age who may enroll in clinical trials involving psychedelics or who may use psychedelics in real-world settings, we conducted a systematic review to synthesize the available evidence on child and maternal health outcomes following antenatal exposure to psychedelics.

## Methods

### Search strategy and information sources

This review followed the *Preferred Reporting Items for Systematic Reviews and Meta-Analyses* (PRISMA) guidelines, and the protocol was pre-registered in PROSPERO (CRD420251082910). The PRISMA checklist for this systematic review is presented in eTable 1 in Supplement 1. A systematic literature search was conducted in Medline, Embase, Web of Science, PsycInfo and CENTRAL on 15 July 2025. All databases were searched from inception without any restrictions on publication year or language. For each search concept, relevant free-text terms and Medical Subject Headings (MeSH) were identified including *pregnancy*, *hallucinogens*, *psilocybin*, *Banisteriopsis, N,N-dimethyltryptamine, lysergic acid diethylamide* and *mescaline*. The full search strategy is presented in eTable 2 in Supplement 1.

### Eligibility criteria

All original peer-reviewed articles were eligible if they reported on pregnant women who were exposed to classic psychedelics during pregnancy. Psychedelics were defined as any of the following substances: psilocybin, ayahuasca, N,N-dimethyltryptamine (DMT), LSD, mescaline and mescaline-containing cacti.^12^ All study designs were eligible for inclusion, including case reports. We did not exclude articles based on context, setting, year of publication, type of article or geographic location. To avoid introducing bias, there was no restrictions on language of article. DeepL and Google Translate were used to translate titles and abstracts, as well as full-text articles. Articles were excluded if they did not include original data, focused only on animal or non-pregnant populations, or reported only on other psychoactive substances such as ketamine, 3,4-methylenedioxymethamphetamine (MDMA), salvia divinorum and ibogaine. These substances were excluded as they are not considered to be classic psychedelics.^12^

### Selection process

All titles, abstracts and full-text articles were independently screened against the eligibility criteria by two of the reviewers (SS, OS, AS, LW, MH), using Covidence systematic review software (Veritas Health Innovation, Melbourne, Australia). Disagreements were resolved through discussion between the two reviewers, with input from a third reviewer where necessary.

### Data collection process

For included studies, two reviewers (from SS, OS, LW, MH) independently performed data extraction and risk of bias assessment for each article. Discrepancies were resolved through discussion between the two reviewers, with input from a third reviewer where necessary. Reviewers used standardized extraction forms to extract the following information: (i) general study information, (ii) study characteristics, (iii) participant information, (iv) exposure, (v) presence of a comparison group, and (vi) reported outcomes. The full data extraction template is available in eTable 3 in Supplement 1.

### Risk of bias assessment, certainty assessment and synthesis method

The Joanna Briggs Institute (JBI) critical appraisal tools were used for study risk of bias assessment (see eTable 4 in Supplement 1).^13^ Certainty assessment was undertaken using the Grading of Recommendations Assessment, Development, and Evaluation (GRADE) approach for each of the primary outcomes: spontaneous abortion, stillbirth, maternal mortality, neonatal mortality, preterm birth, low birthweight and congenital malformation (eTable 5 in Supplement 1).^14^ As significant heterogeneity in study design and outcome reporting precluded meta-analysis, a qualitative synthesis was conducted.

## Results

### Study selection and study characteristics

**Figure 1:**
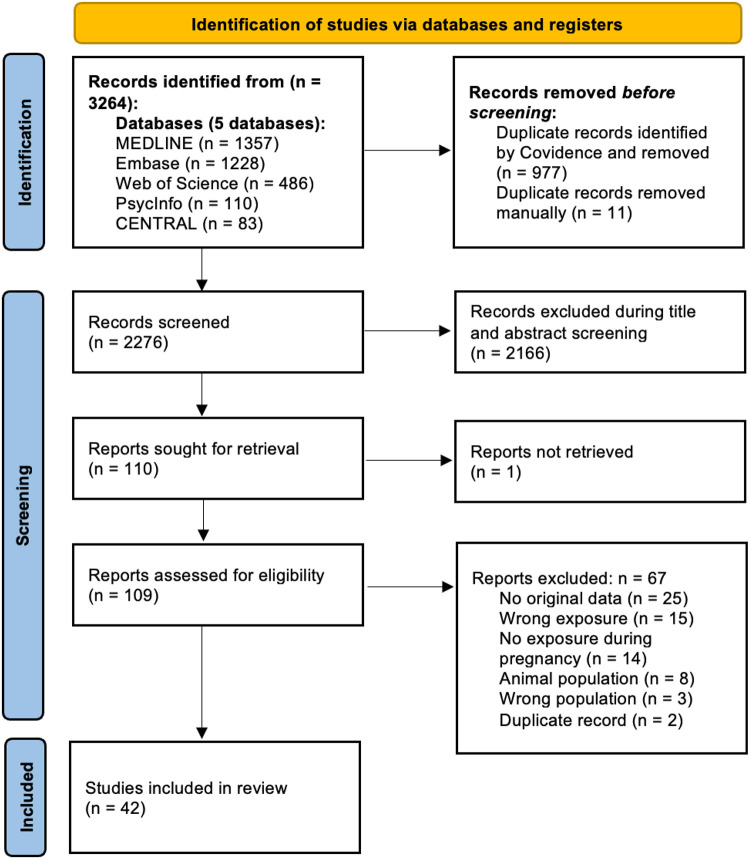
PRISMA flow diagram of the study selection process and exclusion of studies PRISMA = Preferred Reporting Items for Systematic Reviews and Meta-Analyses

A total of 3,264 articles were identified from 5 databases. Following de-duplication, 2,276 titles and abstracts were screened, and 110 articles underwent full-text screening. The PRISMA flow diagram of the study selection process is presented in Figure 1. A total of 42 studies were included in this systematic review, encompassing 251 women who were exposed to a psychedelic substance during pregnancy or prior to pregnancy across nine countries. Almost all studies were conducted in high-income countries (k=38, 90.5%), of which most were conducted in the United States (k=29, 69.0%), including one study conducted in both the United States and the United Kingdom, followed by Canada (k=3, 7.1%), the United Kingdom (k=2, 4.8%) and Australia (k=2, 4.8%). There were 3 studies that were conducted in upper-middle income countries (7.1%), namely Mexico, Brazil and Iran, and no studies were conducted in lowincome or lower-middle income countries.

Most studies were published before the year 2000 (k=31, 73.8%), with the majority conducted between 1960 and 1980 (k=26, 61.9%) and only 4 studies (9.5%) performed since 2015. Case reports (k=23, 54.8%) were the most common study design, followed by cohort studies (k=10, 23.8%), case series (k=4, 9.5%) and cross-sectional studies (k=2, 4.8%). There were 38 studies that reported on LSD exposure during pregnancy, followed by 4 studies (9.5%) reporting on psilocybin, 3 studies (7.1%) reporting on mescaline or mescaline-containing cacti, only 1 study (2.4%) reporting on DMT and 1 study (2.4%) reporting on ayahuasca exposure during pregnancy. Although most studies reported psychedelic exposure in naturalistic settings, two studies reported administration of psychedelic substances by

### Risk of bias in studies and certainty assessment

Of the 42 studies, 28 studies (66.7%) were deemed to be of low to medium risk of bias using the JBI critical appraisal tools and 14 studies (33.3%) were deemed to be of high risk of bias (Table 1). The complete item-level critical appraisal for each study is presented in eTable 4 in Supplement 1. Using the GRADE approach, the certainty of evidence of all outcomes (spontaneous abortions, stillbirth, neonatal mortality, preterm birth, birthweight and congenital malformations) was deemed to be very low due to study design, risk of bias assessment and publication bias (eTable 5 in Supplement 1).

### Reported outcomes

#### Spontaneous abortions

Only two articles reported on spontaneous abortions, both of which were published in the 1970s.^16,38^ Jacobson et al. conducted a cohort study of 148 pregnancies where LSD had been used prior to or during pregnancy and reported 65 abortions (43.9%), of which 12 (8.1%) were spontaneous abortions and 53 (35.8%) were therapeutic abortions performed for psychiatric indications.^38^ In a cohort study by McGlothlin et al., there were 12 pregnancies where LSD had been used during the pregnancy, of which 6 spontaneous abortions were reported, though five were from the same woman.^16^ Neither cohort studies included a non-exposed comparison group. Notably, all women in the Jacobson et al. study consumed marijuana and many of the participants also consumed other drugs, such as amphetamines.

#### Maternal deaths and stillbirth

There were no studies that reported on maternal deaths. Only one case report by Scott et al.^46^ reported on stillbirth in a case of a 20-year-old Australian university student who had been unaware that she was pregnant. She consumed MDMA (not a classic psychedelic) and LSD in the 24 hours prior to the birth of a near-term stillborn infant and subsequently developed eclampsia with hypertension, proteinuria and seizures.

#### Neonatal mortality

A total of 16 articles reported on neonatal mortality or survival, of which 75% were case reports (n=12). Thirteen articles reported on LSD exposure alone. There was one cohort study by Jacobson et al. conducted in 1972 that found one neonatal death (1.2%) out of 83 livebirths amongst pregnancies that had LSD exposure prior to or during pregnancy, however there was no comparison group.^38^ Neonatal death was reported in 3 case reports where there was exposure to LSD during pregnancy.^19,24,32^ Nine articles reported survival during the neonatal period, though of these, 3 case reports reported infant death within the first year of life despite neonatal survival.^21,29,50^

Three studies reported on peyote, ayahuasca and psilocybin exposure during pregnancy.^10,15,53^ Warren et al. documented a case report in 1970 where the infant survived the neonatal period following exposure to both LSD and peyote in utero.^53^ Labate reported that the three children of her interviewee were alive at the time of interview at 5, 13 and 16 years, all of whom had been exposed to ayahuasca in utero.^10^ The interviewee described normal births without complications and described her children as “excellent students” who were “well behaved and sociable.” Leary et al. published a study of an infant who had been exposed to psilocybin during pregnancy at two-week intervals, which had been approved by the mother’s obstetrician. The infant was still alive at one year of age and “no detrimental effects” were reported.^15^

#### Preterm birth and birthweight

Two cohort studies reported on preterm birth.^17,38^ Jacobson et al.^38^ found that of 83 livebirths where there was LSD exposure prior to or during pregnancy, one infant was born premature (1.2%), whilst Aase et al.^17^ reported that 3 out of 10 infants (30.0%) who were exposed to LSD during pregnancy were born preterm. A further 15 articles, consisting of case reports and case series, reported whether the infant was born preterm or at term. A total of 7 articles reported preterm birth whilst the remaining 8 articles reported term birth, though a rate cannot be determined from case reports and case series due to lack of a denominator and inherent publication bias. All articles reporting on preterm birth investigated LSD exposure, with the exception of Warren et al.,^53^ where both LSD and peyote exposure were reported. For the outcome of birthweight, there were 14 case reports and 1 case series where there was antenatal exposure to psychedelics that reported birthweight of the infant. Seven case reports reported low birthweight,^19,22,24,31,39,41,49^ whilst the remaining articles (n=8) reported normal birthweight.

#### Congenital malformations

A total of 26 articles reported on congenital malformations, of which 3 were cohort studies. A cohort study conducted by Jacobson et al. found that 8 out of 83 (9.6%) liveborn infants had major congenital malformations, where LSD had been used prior to or during pregnancy.^38^ Notably, most of these women also reported a history of cannabis or amphetamine use. Conversely, 2 cohort studies of 9 and 10 infants conducted in 1968 and 1970 respectively found no congenital malformations amongst these infants following antenatal exposure to LSD.^17,26^ Of the case reports that reported the presence of a congenital malformation, the majority reported psychedelic exposure during the first trimester, which is known to be a critical period for fetal organ development. In terms of categories of congenital malformations, 8 articles reported limb anomalies, 7 reported eye anomalies, 6 reported nervous system anomalies, 4 reported congenital heart defects, 4 reported gastrointestinal anomalies, 3 reported kidney and urinary tract anomalies and 3 reported oro-facial clefts (eTable 6 in Supplement 1).

## Discussion

We conducted the most comprehensive systematic review to date on child and maternal health outcomes following antenatal exposure to classic psychedelic substances. The findings highlight several limitations in the existing literature, including small samples drawn almost exclusively from high-income countries, study designs that were either inherently limited or weakened by high risk of bias, and an overrepresentation of older studies on LSD. It underscores the urgent need for contemporary, methodologically rigorous research on psychedelic exposure during pregnancy.

There were 42 studies included in this systematic review, most of which were from the United States and other high-income countries. In these studies, 251 women were reported to have been exposed to psychedelics prior to or during pregnancy, which is considerably smaller than the evidence base available for other substances used during pregnancy, such as cannabis.^55^ Notably, the majority of studies that were included in this systematic review were case reports, which represent low quality evidence. While there were studies with arguably stronger study designs (e.g., cohort studies), many of these were deemed to have moderate or high risk of bias. Furthermore, most of the cohort studies lacked an appropriate comparison or unexposed group, precluding causal inferences between antenatal psychedelic exposure and perinatal outcomes. There was also limited information on dose and co-use of other substances, which are important predictors of outcomes in perinatal epidemiology.^56,57^ Such limitations make it difficult to establish a causal relationship between psychedelic exposure during pregnancy and child and maternal health outcomes. This is especially relevant as epidemiological research has long suggested strong associations between psychedelic use and other substance use known to be harmful during pregnancy, such as tobacco and alcohol.^58,59^

Although several articles reported on congenital malformations, preterm birth and neonatal mortality, very few articles reported on spontaneous abortions and stillbirth, and no articles reported on maternal deaths. Limb anomalies, eye anomalies and nervous system anomalies were the most commonly reported congenital malformation categories, though these were mainly reported through case reports, and were thus particularly susceptible to publication bias, reporting bias, selection bias, and confounding. Incidence cannot be ascertained due to absence of a denominator as the total number of exposed individuals remains unknown. Nonetheless, these reports warrant further investigation into the potential impact of psychedelic substances on congenital malformations. Interestingly, most studies that documented timing of psychedelic exposure reported that exposure occurred during the first trimester, a window of time that is known to be critical for fetal organogenesis.^60,61^ This is also often a time when women may be unaware that they are pregnant, which was reported in several of the included studies.^31,34,46,52,53^ This is of particular contemporary relevance, as there is accelerated growth in the use of psychedelics, especially amongst younger adults, where rates of unintended pregnancies are high.^2,62,63^

The majority of studies included in this systematic review were conducted in the 1960s and 1970s, a period in which scientific and cultural interest was primarily focused on LSD.^64^ However, psilocybin has now become the most commonly used psychedelic substance in the United States,^65^ while other psychedelics are often used during pregnancy in certain communities for cultural and religious reasons, such as ayahuasca consumption amongst the Santo Daime communities in Brazil, and peyote ingestion amongst Indigenous peoples in North America and the Wixárika people of Mexico.^9–11^ Despite this, our systematic review only identified 8 articles that reported on psychedelic substances other than LSD: 4 articles on psilocybin, 3 articles on mescaline or mescaline-containing cacti (such as peyote) and only 1 article each on DMT and ayahuasca. It should therefore be a priority to expand the evidence base to include a broader range of psychedelic substances, particularly given the possibility of differential effects on fetal development across substances with distinct pharmacological profiles. Furthermore, communities that consume psychedelic substances for cultural or religious purposes may represent an understudied group who may have increased rates of psychedelic exposure during pregnancy. However, research involving these populations should employ community-based participatory research frameworks to ensure cultural sensitivity, meaningful engagement, and feasibility.^66^

Of all the studies included in the systematic review, Jacobson et al.’s cohort study had the largest number of women exposed to psychedelics prior to or during pregnancy.^38^ Notably, of 83 live newborns, 9.6% had major congenital malformations of varying types, which is higher than general population estimates reported in the literature.^67–71^ It is important to note, however, that the study did not have an unexposed comparison group and the study included women who reported exposure to LSD before or during pregnancy, without stratifying outcomes by whether exposure occurred before or during pregnancy. Furthermore, the majority of these women also reported a history of cannabis or amphetamine use – substances that may be associated with congenital malformations.^72–74^ No reporting was made on whether these substances were used during pregnancy, though, further complicating the findings. Taken together, the Jacobson et al. cohort study includes limitations that are common across the included studies.^16,17,25^

Clinicians should be aware of the paucity of robust evidence and safety data for antenatal exposure to psychedelic substances and counsel pregnant women and women of reproductive age accordingly. Given that there is growing scientific and public interest in psychedelics as a potential treatment for a range of psychiatric conditions, this highlights a critical research gap. Future research should focus on rigorous prospective cohort studies for a range of psychedelic substances beyond LSD, include data from low- and middle-income countries, use an appropriate comparison group, adjust for important confounders, and consider understudied communities that may have a higher prevalence of antenatal exposure to psychedelic substances due to cultural and religious reasons. To allow for future meta-analysis on this topic, researchers should report on the psychedelic exposure and timing (before or during pregnancy, ideally gestational age of exposure), means of exposure assessment, other substance use and timing, substance use history, and whenever possible dose of exposure, context of use, and awareness of pregnancy at time of exposure. Pregnancy outcomes should be objectively measured. Future work should include expansion of other non-classic psychedelics and their effects on pregnancy outcomes as well.

## Conclusions

This systematic review highlights a discrepancy between the growing use of psychedelics and the quality of evidence available to inform clinical guidance. The existing literature is limited and ultimately insufficient to establish if and how psychedelic exposure during pregnancy may represent a risk to child and maternal health. As the cultural, scientific, and legal landscape surrounding psychedelics continues to shift toward greater use and acceptability, studies that address the limitations identified in this systematic review are urgently needed to support evidence-based counselling for pregnant women exposed to psychedelics, those planning pregnancy while using psychedelics, or reproductive-age women enrolled in psychedelic clinical trials.

## Supplementary Material

Supplementary Files

This is a list of supplementary files associated with this preprint. Click to download.

- Supplement1v16.05.26.docx

## Figures and Tables

**Table 1.** Characteristics of included studies

Study ID	Country	Study design	Total sample size	Total number of pregnant women exposed to a psychedelic substance	Type of psychedelic substance exposure during pregnancy	Ascertainment of psychedelic exposure	Timing of exposure to psychedelic substance	Risk of bias
**Aase 1970** ^17^	US	Cohort study	20	10	LSD	Self-reported	First trimester; second trimester; third trimester	Low
**Aleguas 2015** ^18^	US	Case report	4	1	LSD	Objective	Third trimester	Moderate
**Apple 1974** ^19^	US	Case report	1	1	LSD	Self-reported	Not specified	Moderate
**Assemany 1970** ^20^	US	Case report	1	1	LSD	Self-reported	Not specified	Moderate
**Bogdanoff 1972** ^21^	US	Case report	1	1	LSD	Self-reported	Not specified	Low
**Carakushansky 1969** ^22^	US	Case report	1	1	LSD	Self-reported	Not specified	Moderate
**Carter 1972** ^23^	US	Case series	20	5	LSD	Self-reported	Not specified	High
**Chan 1978** ^24^	US	Case report	1	1	LSD	Self-reported	First trimester	Moderate
**Cohen 1967** ^25^	US	Case series	42	3	LSD	Self-reported	First trimester; second trimester; third trimester	High
**Cohen 1968** ^26^	US	Cohort study	34	7	LSD	Self-reported	First trimester; second trimester; third trimester	High
**Egozcue 1968** ^27^	US	Cohort study	64	4	LSD	Self-reported	First trimester; second trimester; third trimester	High
**Egozcue 1969** ^28^	Not reported	Cross sectional study	20	6	LSD	Self-reported	First trimester; second trimester; third trimester	Moderate
**Eller 1970** ^29^	US	Case report	1	1	LSD	Self-reported	First trimester; around time of conception	Low
**Faria 1973** ^30^	US	Case report	38	1	LSD	Self-reported	First trimester	Moderate
**Gelehrter 1970** ^31^	US	Case report	1	1	LSD	Self-reported	First trimester	Low
**Giovannucci 1976** ^32^	Italy	Case report	2	1	LSD	Self-reported	Not specified	Moderate
**Gómez-Ruiz 2022** ^33^	Mexico	Cohort study	300	1	DMT	Objective	Not specified	Moderate
**Haden 2020** ^34^	Canada	Case report	3	1	LSD	Interview with the pregnant woman and the supplier of the LSD	First trimester	High
**Hirschhorn 1968** ^35^	US	Case series	34	4	LSD	Self-reported	Not specified	Moderate
**Ho 2001** ^36^	Canada	Cross sectional study	254	11	Psilocybin, LSD, mescaline	Self-reported	First trimester; second trimester	Moderate
**Hoyt 1978** ^37^	US	Case series	2	2	LSD	Self-reported	First trimester; second trimester; third trimester	High
**Jacobson 1972** ^38^	US	Cohort study	140	140*	LSD	Self-reported	First trimester; second trimester; third trimester	High
**Jeanbart 1971** ^39^	Canada	Case report	1	1	LSD	Self-reported	First trimester	Moderate
**Kelly 2000** ^40^	Australia	Case report	296	1	LSD	Self-reported	Not specified	High
**Labate 2011** ^10^	Brazil	Qualitative research	1	1	Ayahuasca	Self-reported	Not specified	Moderate
**Leary 1963** ^15^	US	Non-randomized experimental study	175	1	Psilocybin	Objective	Not specified	High
**Margolis 1980** ^41^	US	Case report	1	1	LSD	Self-reported	Not specified	Low
**McElhatton 1999** ^42^	UK	Cohort study	127	9	LSD	Self-reported	Not specified	High
**McGlothlin 1970** ^16^	US	Cohort study	247	12	LSD	Objective and self-reported (LSD administered medically, but there was also non-medical use that was self-reported)	Not specified	High
**Moore 2010** ^43^	UK	Cohort study	86	5	Psilocybin, LSD	Self-reported	First trimester	High
**Saeidi 2016** ^44^	Iran	Cohort study	100	1	LSD	Self-reported	Not specified	High
**Sato 1968** ^45^	US	Case report	1	1	LSD	Self-reported	First trimester	Moderate
**Scott 2010** ^46^	Australia	Case report	1	1	LSD	Self-reported, though confirmed with urine drug screen	First trimester; second trimester; third trimester	Low
**Singer 2012** ^47^	US and UK	Cohort study	96	7	Psilocybin, LSD	Self-reported	Not specified	Low
**Stenchever 1970** ^48^	US	Case report	21	1	LSD	Not specified	Second trimester	High
**Tenbrinck 1975** ^49^	US	Case report	1	1	LSD	Self-reported	First trimester	Moderate
**Tenorio 1988** ^50^	US	Case report	1	1	LSD	Self-reported	First trimester; second trimester	Low
**Torfs 1994** ^51^	US	Case control study	330	2	LSD	Self-reported	First trimester; pre-conceptional trimester	Low
**Van Tol 2009** ^11^	US	Case report	1	1	Peyote	Report from nurse and father	During labor	Low
**Von Mandach 1999** ^52^	Switzerland	Case report	1	1	LSD	Self-reported	First trimester	Moderate
**Warren 1970** ^53^	US	Case report	1	1	LSD, peyote	Self-reported	First trimester; second trimester	Moderate
**Zellweger 1967** ^54^	US	Case report	1	1	LSD	Self-reported	First trimester; second trimester	Moderate

* Includes women who were exposed to a psychedelic substance during pregnancy and/ or prior to pregnancy.

DMT = N,N-dimethyltryptamine, LSD = lysergic acid diethylamide, UK = United Kingdom, US = United States

## Data Availability

This systematic review extracted data from published articles. Extracted data are summarized in the article and supplement and are available from the corresponding author upon reasonable request.

## Key points

### Question:

What are the child and maternal health outcomes reported following antenatal exposure to psychedelics?

### Findings:

This systematic review included 42 studies cross 9 countries. Although there were some articles that reported on outcomes such as congenital malformations and preterm birth, most studies had small samples, used study designs that are inherently susceptible to bias (54.8% were case reports), only focused on LSD exposure, and were conducted prior to 2000.

### Meaning:

The evidence on outcomes following psychedelic use during pregnancy is sparse and of low quality. Robust research to ascertain outcomes following antenatal exposure to psychedelics is urgently needed.
